# Systemic fungal infection in a dog: a unique case in Ireland

**DOI:** 10.1186/2046-0481-67-17

**Published:** 2014-08-06

**Authors:** Sabela Atencia, Stratos Papakonstantinou, Bernadette Leggett, Hester McAllister, Carmel T Mooney

**Affiliations:** 1 Department of Small Animal Clinical Studies, University Veterinary Hospital, University College Dublin, Belfield, Dublin Dublin 4, Ireland; 2 Department of Diagnostic Imaging, University Veterinary Hospital, University College Dublin, Belfield, Dublin Dublin 4, Ireland; 3 Department of Microbiology, University Veterinary Hospital, University College Dublin, Belfield, Dublin Dublin 4, Ireland; 4 Biobest Laboratories Ltd. 6 Charles Darwin House, The Edinburgh Technopole, Milton Bridge, Nr Penicuik EH26 0PY, Scotland

**Keywords:** Fungus, Dog, Systemic infection, Ciclosporin

## Abstract

A three year old male entire Staffordshire bull terrier was referred to University College Dublin Veterinary Hospital, with a two week history of fever, inflammation of the right hock, lameness on the right hindlimb, peripheral lymphadenopathy and gastrointestinal signs (vomiting and diarrhoea). For the preceding three months the dog had been treated for atopic dermatitis with oral ciclosporin (5 mg/kg, PO, q 24 hours).

Cytological analysis of the affected lymph nodes demonstrated fungal-like organisms predominantly contained within macrophages. Subsequent fungal culture and microscopic identification confirmed the presence of a *Byssochlamys sp*. This fungus is a saprophytic organism which has been associated with mycotoxin production. It has not previously been identified as a cause of systemic infection in animals or humans.

Ciclosporin was discontinued, and a second generation triazole, voriconazole prescribed at a dose of 6 mg/kg for the first two doses, and continued at 3 mg/kg every 12 hours for six months. There was an excellent response. Follow-up examination five weeks after treatment was completed confirmed remission of the disease. The dog remains alive and well three years later.

The present case represents an unusual fungal infection in a dog secondary to immunosuppressive therapy with ciclosporin. Such a possibility should be considered in animals presenting with signs consistent with systemic infection when receiving immunosuppressive medication.

## Background

The immunosuppressive effects of ciclosporin have the potential to result in secondary bacterial [1,2], fungal [3], or parasitic [4,5] infections or malignancy [6,7]. Recently a systemic fungal infection in a dog treated with immunosuppressive therapy with ciclosporin was described [8].

The present report is the first documented case of a systemic fungal infection with a *Byssochlamys sp* in a dog that had been receiving chronic immunosuppressive therapy with ciclosporin. Previously associated with mycotoxin production, *Byssochlamys sp* has not previously been identified as a cause of systemic infection in animals or humans.

## Case report

A three year old male entire Staffordshire bull terrier presented to the University College Dublin Veterinary Hospital with a two week history of pyrexia, gastrointestinal signs (vomiting and diarrhea), oedematous swelling of the right hind limb around the hock and moderately enlarged right pre-scapular and right popliteal lymph nodes. All other peripheral lymph nodes were within normal limits. The referring veterinarian had initiated therapy with oral cephalexin which had not resulted in any significant improvement.

Three months prior to presentation, the dog had been treated for suspected atopic dermatitis with immunosuppressive therapy (ciclosporin 5 mg/kg, q 24 hours with prednisolone at 1 mg/kg, q 24 hours). At the time of presentation the dog was still receiving daily ciclosporin. The prednisolone had been discontinued one month prior to the onset of clinical signs. Ciclosporin was discontinued the day of admission to the hospital.

On physical examination the dog was lethargic and pyrexic (40.1°C). The right pre-scapular and right popliteal lymph nodes were palpably and moderately enlarged. The dog was non weight bearing on the right hindlimb, oedematous swelling of the right hock was detected, without evident joint effusion. No abnormalities were noted on palpation of the abdomen or on thoracic auscultation.

Haematological examination demonstrated mature neutrophilia (13.06 (reference interval, 3–11.5) × 10^9^/L). Serum biochemistry identified hyperglobulinaemia (55.8 (reference interval, 28–42) g/L) and hypoalbuminaemia (20.1 (reference interval 25–40) g/L). Proteinuria (3+) was present on urinalysis, with a urine protein:creatinine ratio of 6.4 (reference interval < 0.5). The urinary sediment revealed a few casts and occasional white blood cells. Bacterial culture was negative. Serum protein electrophoresis showed mild polyclonal gammopathy, indicative of chronic antigenic stimulation. The rest of the biochemistry was within reference limits.

**Figure 1:**
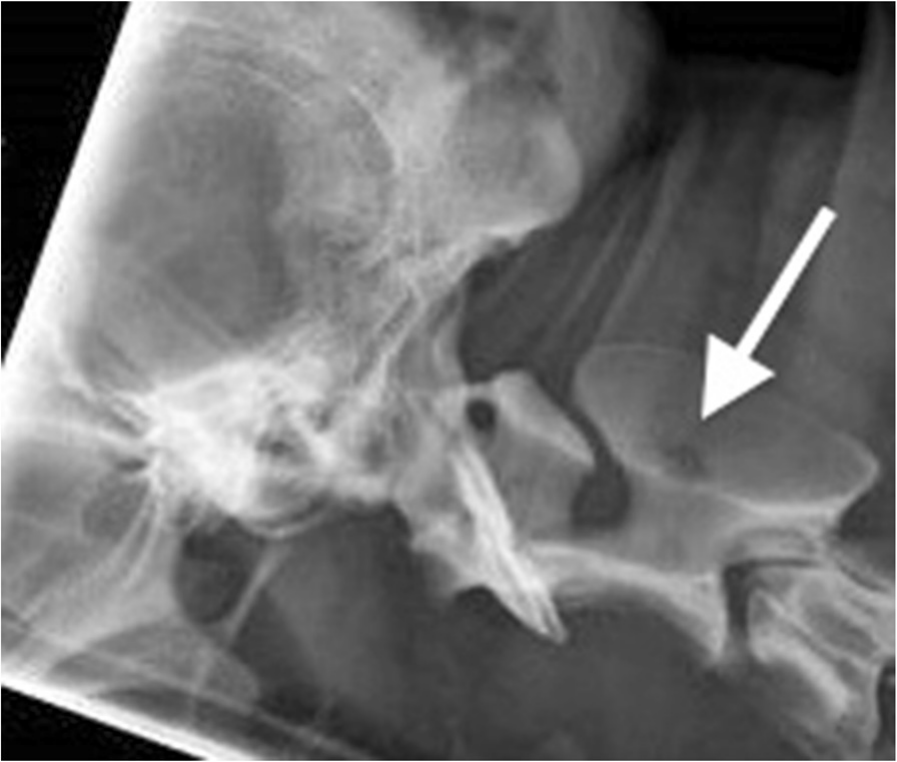
**Lateral radiograph of the cervical spine.** A focal circular osteolytic area is present within the dorsal arch of the axis (arrow).

**Figure 2:**
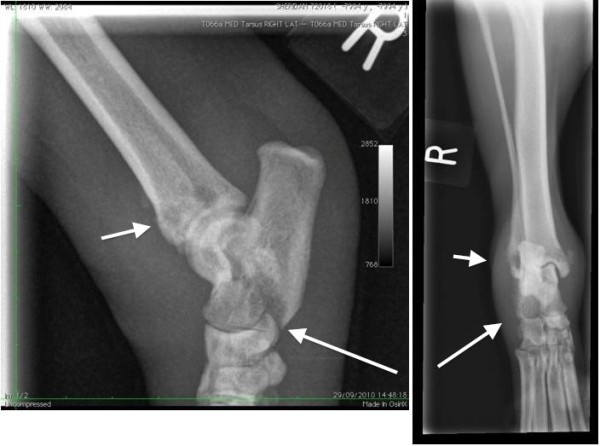
**Mediolateral and dorsoplantar radiographs of the right tarsocrural joint.** There are focal areas of osteolysis in the distal 1cm of the tibial diaphysis, distal fibula (short arrows) and the plantarodistal aspects of the body of the calcaneus. (long arrows). Proliferative active periosteal bone formation is present along the lateral aspect of the calcaneus and distomedial tibial diaphysis and cranio distal tibial diaphysis. A large soft tissue swelling encircles the joint.

Radiographs of the right hock and thoracic spine showed focal areas of osteolysis and new bone formation within the dorsal arch of the axis, and in the distal 1 cm of the tibial diaphysis, distal fibula and the plantarodistal aspects of the body of the calcaneus (Figures 1 and 2). These findings were suggestive of a neoplastic or infectious process.

The spleen and the iliac lymph nodes were mildly enlarged but had normal echogenicity on the abdominal ultrasonographic examination. Ultrasound guided fine needle aspirations (FNAs) were taken from the spleen and iliac lymph nodes. On cytological examination there was a non-septic neutrophilic inflammation, with no signs of malignancy.

**Figure 3:**
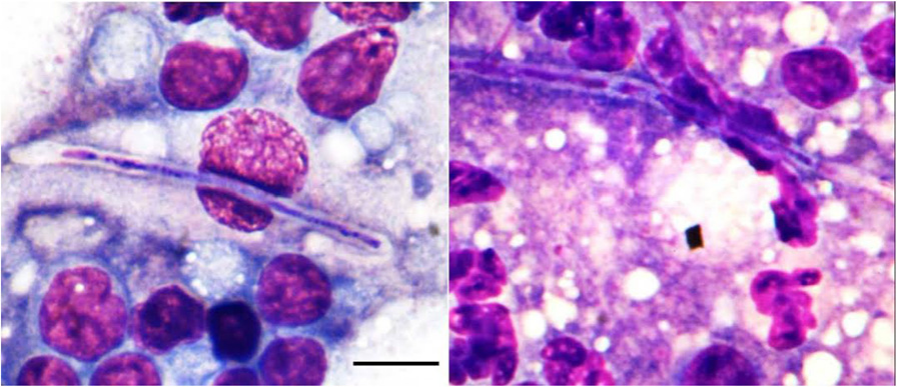
**Smears of fine needle aspirates of the right pre-scapular and popliteal lymph nodes were air dried and then fixed in methanol and stained using an automated slide-stainer (Westcor Aerospray Haematology Stat Slide Stainer, Westcor, Logan, Utah) and a two-part Romanowsky stain with eosin and thiazine.** Typical appearance of fungal hyphae in peripheral lymph nodes. The fungal hyphae are found in a light basophilic to eosinphilic background and surrounded by a mixture of large macrophages, small-sized lymphocytes, and moderately-degenerate neutrophils. The hyphae are 30 – 50 um long by 3-4 um wide (scale bar (10 um)), occasionally with faint septae visible, without branching, and with mixed staining of the interior.

FNAs of the oedematous area affecting the right hock were attempted, but the cytology was non diagnostic given the poor cell yield. Samples of the right carpo-tarsal joint were not taken pending FNA results from the enlarged lymph nodes (right prescapular and popliteal). Lymph node smears showed moderate plasma cell hyperplasia and mild pyogranulomatous inflammation in association with fungal hyphae. Smears were highly cellular in a light background of fresh blood. Nucleated cells were predominantly lymphocytes, most of which were smaller in size than a neutrophil and had only scant cytoplasm. There were frequent plasma cells with prominent cytoplasmic basophilia and perinuclear clearing zones, occasionally binucleate. Rarely, fungal hyphae were seen, associated with increased numbers of neutrophils (mildly degenerate) and with macrophages. The hyphae were long, linear structures, up to 50 um in length and 3 to 4 um in width, without significant branching. Most of the hyphae were unstained, although the central one-third had dark, mixed, internal staining. Occasionally, septae were seen, with length of 10 – 15 um. No bacteria were seen. There were occasional, solitary mast cells. These findings were suspicious of mycosis (Figure 3). Aspirates of the affected lymph node were submitted for bacterial and fungal culture.

Ultrasound guided biopsies, using a semi-automatic Bard ®Magnum Reusable tru-cut Core Biopsy System device, were taken from the right pre-scapular lymph node, and were submitted for histopathology and bacterial and fungal culture. Histopathology depicted a reactive lymph node with no signs of malignancy or fungal elements.

A jugular blood sample (10 milliliters), FNA from the right prescapular and popliteal lymph node, and a tissue sample of the right pre-scapular lymph node were submitted for further investigations. The blood sample was inoculated into a blood culture system (Oxoid, Basingstoke, U.K.) and incubated at 37°C for 7 days. The FNA and tissue samples were routinely cultured using Columbia Blood Agar (Oxoid, Basinstoke, U.K.) enriched with 5% sheep’s blood (Cruinn Diagnostics, Ireland), Columbia Blood Agar supplemented with colisitn-nalidixic acid (Oxoid, Basingstoke, U.K.) and MacConkey agar number 2 (Oxoid, Basingstoke, U.K.). Plates were incubated under aerobic and anaerobic conditions up to 36 hours in the event of no visible growth at 18 hours. Sabouraud Dextrose Agar plates (Oxoid, Basingstoke, U.K.) were also inoculated and incubated at 25 and 37°C for 5 days for the detection of pathogenic fungi or yeast. Direct Gram and Methylene Blue stains from the FNA and tissue samples were carried out. No organisms were observed from the prepared slides.

**Figure 4:**
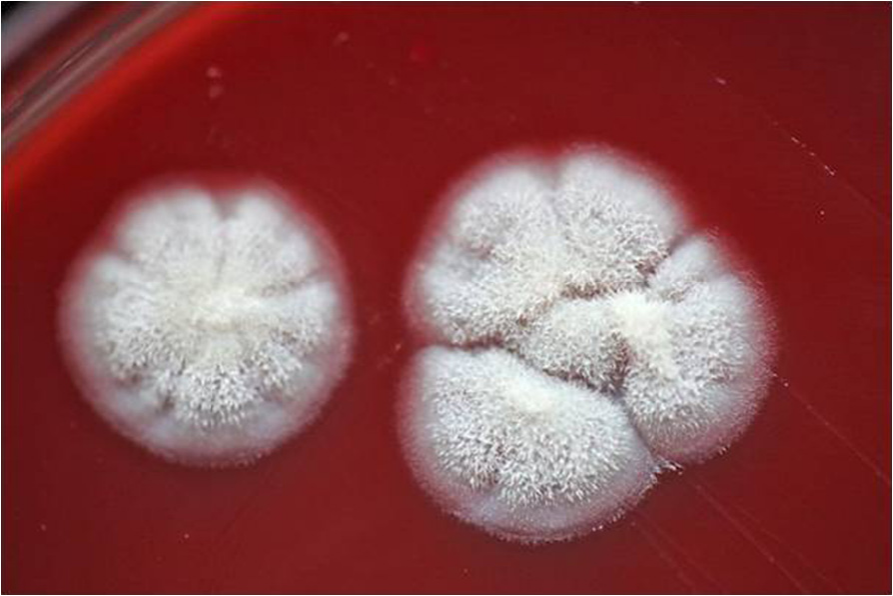
**Two fungal colonies growing on Sabouraud dextrose agar.** The colony morphology under 25 degrees was small white cotton like. Under 37 degrees the colonies were waxy and yeast like, they were cream in colour. Under the microscope fungal hyphae were seen with some oval conidia.

Bacterial cultures were examined for growth after 18 and 36 hours and a negative result was recorded. A negative result was recorded for the blood culture after 7 days. The fungal cultures were examined after 5 days. The FNA fungal cultures were negative, however two fungal colonies were isolated from the prescapular lymph node tissue sample (Figure 4).

A smear of this isolate was prepared and stained with Methylene Blue. Hyphal structures were observed under the microscope. The sample was sub-cultured and sent to The Mycology Reference Laboratory (Myrtle Road, Bristol, U.K.) for identification. The isolate was identified as a probable *Byssochlamys sp..* The identification was based upon colonial appearance and microscopic morphology.

Treatment commenced with itraconazole 5 mg/kg, PO, q 24 hours (Itrafungol, Janssen) and clindamycin 5 mg/kg, PO, q 12 hours (Antirobe, Pfizer), while the culture results were pending. Tramadol hydrochloride 3 mg/kg, PO, q 12 hours (Zydol, Grunenthal) and gabapentin 10 mg/kg, PO, q 12 hours (Neurotonin, Pfizer) were prescribed for analgesia. Opioid analgesia with fentanyl patches (50ug/hour) was also utilized.

After 10 days of hospitalization there was no clinical improvement. Voriconazole (Vfend, Pfitzer) at 6 mg/kg, PO for the first 2 doses, followed by 3 mg/kg, PO, q 12 hours was introduced. Itraconazole and clindamycin were discontinued at that time. Two episodes of vomiting were noted at the initiation of therapy with voriconazole, after which no other side effects were noted.

The dog’s condition dramatically improved over the first 7 days on voriconazole. The body temperature normalized, the lameness improved, and the swelling of the right hock diminished.

Antifungal treatment with voriconazole was continued for a total of 6 months. The progression of the disease was followed up monthly by repeating FNAs with cytology and culture of the affected lymph nodes. The swelling of the right hock had completely resolved 26 days after voriconazole was started. Fungal organisms were detected on cytology up until the 4^th^ month on treatment. All subsequent samples yielded negative fungal culture results. Six months after therapy was started the cytology of two consecutive FNAs (spaced one month apart) were also free of fungi, and the treatment was discontinued. The dog is still alive at the time of writing (3 years after diagnosis), and free of clinical signs.

## Discussion

Systemic fungal diseases cause significant morbidity and mortality in dogs and cats. The species involved are usually opportunistic and frequently affect immunocompromised animals. Infections disseminate from a single portal of entry, either by inhalation, direct wound contamination or ingestion [9,10]. In the current case the portal of entry was most likely oral as there was no history or obvious evidence of a penetrating wound or respiratory involvement, however a percutaneous route of entrance with secondary haematogenous spread could not be excluded [9].

Ciclosporin has traditionally being used in humans, cats and dogs undergoing transplantation surgery. Other recent uses include medical therapy for anal furunculosis, autoimmune diseases and treatment of atopic dermatitis in the dog [11,12]. In Ireland ciclosporin is only licensed for treatment of chronic manifestations of atopic dermatitis in dogs. There are sporadic reports of dogs and cats developing secondary fungal, bacterial or parasitic infections after immunosuppressive treatment with ciclosporine [1-5]. Its immunosuppressive effects can also result in secondary malignancy [6,7]. Serum ciclosporin concentration is considered the best method currently available to assess adequacy of treatment, however it is normally reserved for patients that fail to respond to standard treatment [12]. Although it may have been interesting to measure circulating ciclosporin concentrations it was not considered necessary as it was discontinued on admission. However, it is likely that the immunosuppressive therapy with ciclosporin played a major role in the development of the fungal infection in the dog in this report.

The case findings were reported to the manufacturer of ciclosporin (Novartis), the Veterinary Medicine Directorate (VMD) and the Irish Medicines Board (IMB). Following the authors’ query, the latter two organizations reported that a limited number of localized bacterial and fungal infections in animals being treated with ciclosporin existed in their respective databases. VMD had recorded a case of toxoplasmosis in one cat and both FIV and FeLV in another cat following ciclosporin treatment. Other side-effects reported to the IMB included diabetes mellitus, emesis, pancreatitis, hypersensitivities, lymphadenopathy and limb weakness. Gingival hyperplasia and papillomas have also been reported [11,12]. As with most other immunosuppressive agents, the manufacturer states that it may increase susceptibility to secondary infections. However to date the VMD and IMB have not received any reports of systemic bacterial or fungal infections following use of ciclosporin despite the existence of a case report in a dog in the UK [8]. The dog of that report had similarly been receiving immunosuppresive therapy with oral ciclosporin [8]. The dog was initially treated with a combination of terbitafine and itraconazole but despite this treatment the clinical signs persisted, and a second generation triazole, voriconazole, was started in combination with terbitafine. This therapy was continued for a year and since then no episodes of recurrence of any clinical signs were reported. The long-term dose of voriconazole applied in this case (3 mg/kg every 12 hours) was slightly below that recommended in the literature (4 mg/kg every 12 hours) [13]. The lower dose was chosen because of the limited size of tablets available (50 or 200 mg) and a reluctance to split them to achieve a dose of 4 mg/kg. Additionally because of financial constraints only a 6 month course was administered. Despite this, complete resolution was documented with serial fine needle aspirates from the affected lymph node.

There are a few limitations in the present study. The exact fungal species was not identified. However given the knowledge of the genus involved further identification would not have altered the therapeutic decisions made. Susceptibility studies were not performed but given the excellent response to empirical treatment with voriconazole, susceptibility is assumed. On the other hand, itraconazole appeared to have a limited effect. Whether this can be translated to all fungi within the genus is not clear. The length of time therapy should be continued is unclear and few definitive recommendations exist in the literature. In the current case, therapy was continued until two consecutive FNAs, spaced one month apart, were negative and this methodology appeared to provide an excellent result. Interestingly fungal culture results were consistently negative whilst on treatment. Although this is a plausible scenario, it does emphasize that reliance should not be placed solely on culture results to guide therapeutic efficacy.

It would have been interesting to see if the magnitude of hyperglobulinaemia decreased after treatment, but given the clinical improvement of the dog, the excellent response to the treatment, and the owner’s financial constraints, these biochemical changes were not re-evaluated.

Proteinuria was detected in the current case during initial investigations. Whilst this could be due to post renal disease, bacterial urine culture was negative. Given the magnitude of the proteinuria, the clinical history and clinicopathological findings, functional renal proteinuria was considered a possibility [14]. However a previous report stated that physiological proteinuria as a result of fever is typically lower than in the present case (UPCR < 0.5) [15]. On the other hand, although fungal elements were not observed on examination of the urine, specific fungal urine culture was not carried out, and consequently the possibility of renal fungal infection cannot be completely excluded. Unfortunately further investigation of the proteinuria by repeating urine protein creatinine ratios were not performed.

The main differential diagnoses for the multifocal osteolytic changes observed included neoplastic, metabolic/endocrine or infectious disease processes. Given the results of protein electrophoresis, multiple myeloma was effectively excluded. Metabolic/endocrine causes were considered unlikely given the lack of other supportive clinical signs or clinicopathological abnormalities. Infectious causes were therefore considered to be a priority. The oedema of the right hock was most likely due to increase in vascular permeability (either due to infectious, or inflammatory processes); other differentials such as venous obstruction or compression were considered less likely after the clinical examination. Initially, given that fungal infections are not endemic in Ireland, greater focus was placed on possible bacterial infection. The results of the lymph node FNA was however highly suggestive of fungal infection prompting the performance of specific fungal cultures.

## Conclusion

This report provides an example of a systemic fungal infection in a dog from Ireland receiving immunosuppressive ciclosporin treatment. There was a successful response to voriconazole administration. Systemic fungal infection should always be considered as a potential complication of immunosuppressive therapy with ciclosporin even in areas where fungal infections are not usually considered endemic. Reporting adverse effects of any drug to the relevant national bodies is necessary in order to ensure adequate and accurate information is amassed.

## Competing interests

The authors declare that they have no competing interests.

## Authors’ contributions

SA was the primary clinician in charge of the case and drafted the manuscript. CTM supervised the case and helped SA in the decision making. HMc performed the imaging studies. BL performed the microbiological and fungal cultures, and SP identified the fungus in the cytology. All authors contributed in the writing of their respective sections, and they all read and approved the final manuscript.
